# Revised Taxonomy and Expanded Biodiversity of the Phytomyxea (Rhizaria, Endomyxa)

**DOI:** 10.1111/jeu.12817

**Published:** 2020-11-08

**Authors:** Michaela Hittorf, Susanne Letsch‐Praxmarer, Alexandra Windegger, David Bass, Martin Kirchmair, Sigrid Neuhauser

**Affiliations:** ^1^ Institute of Microbiology University of Innsbruck Technikerstr. 25 6020 Innsbruck Austria; ^2^ Department of Life Sciences The Natural History Museum Cromwell Road London SW7 5BD United Kingdom; ^3^ Centre for Environment, Fisheries and Aquaculture Science (Cefas) Barrack Road, The Nothe Weymouth DT4 8UB United Kingdom

**Keywords:** eDNA, freshwater, *Hillenburgia*, *Ligniera verrucosa*, moss, *Ostenfeldiella*, plant pathogen, plasmodiophorids, *Pseudolingniera*

## Abstract

Phytomyxea (phytomyxids) is a group of obligate biotrophic pathogens belonging to the Rhizaria. Some phytomyxids are well studied and include known plant pathogens such as *Plasmodiophora brassicae*, the causal agent of clubroot disease. Despite this economic importance, the taxonomy and biodiversity of this group are largely cryptic, with many species described in the premolecular area. Some of these species were key for establishing the morphotaxonomic concepts that define most genera to this day, but systematic efforts to include and integrate those species into molecular studies are still lacking. The aim of this study was to expand our understanding of phytomyxid biodiversity in terrestrial environments. Thirty‐eight environmental samples from habitats in which novel and known diversity of Phytomyxea was expected were analysed. We were able to generate 18S rRNA sequences from *Ligniera verrucosa*, a species which is well defined based on ultrastructure. Phylogenetic analyses of the collected sequences rendered the genera *Lignera*, *Plasmodiophora* and *Spongospora* polyphyletic, and identified two novel and apparently diverse lineages (clade 17, clade 18). Based on these findings and on data from previous studies, we formally establish the new genera *Pseudoligniera* n. gen. for *L. verrucosa*,*Hillenburgia* n. gen. for *Spongospora nasturtii* and revert *Plasmodiophora diplantherae* to its original name *Ostenfeldiella diplantherae*.

PHYTOMYXEA (phytomyxids) is a group of obligate biotrophic plant or stramenopile pathogens and is subdivided into the marine Phagomyxida and terrestrial Plasmodiophorida. For a long time, their taxonomic status was unclear and many researchers considered them as fungi or fungal‐like organisms (Sparrow 1960; Waterhouse 1973). Today, molecular studies place the phytomyxids robustly within the eukaryote supergroup Rhizaria (Bass et al. 2009; Bass et al. 2005; Cavalier‐Smith and Chao 2003). Only a few species with substantial economic impact are well studied (Bulman and Braselton 2014; Neuhauser and Kirchmair 2009; Schwelm et al. 2018). *Plasmodiophora brassicae*, the cause of clubroot disease in brassicas, is the economically most important disease of brassica crops (Dixon 2009; Laila et al. 2019; Wang et al. 2019). Another relatively well‐studied example of a phytomyxid that directly causes economic losses is *Spongospora subterranea*, the cause of the powdery scab disease in potatoes (Balendres et al. 2016; Falloon et al. 2016; Merz and Falloon 2009). Some plasmodiophorids have been identified as vectors for viruses: *Spongospora nasturtii* causes the crook root disease in watercress and transmits the Watercress Yellow Spot Virus (Grenville and Clarkson 2002; Tamada and Kondo 2013), *S. subterranea* is a vector for the Potato Mop‐Top Virus (PMTV) (Beuch et al. 2015; Ciaghi et al. 2018; Tamada and Kondo 2013), and *Polymyxa graminis* transmits several viruses, like the Soil‐Borne Wheat Mosaic Virus (SBWMV) or the Peanut Clump Virus (PCV) (Dieryck et al. 2011; Kanyuka et al. 2003; Tamada and Kondo 2013).

To date, Phytomyxea include 42 species in twelve genera (Murúa et al. 2017; Neuhauser et al. 2011a; Neuhauser et al. 2011b). For 14 phytomyxid species, sequences are available, of which nine are placed within the plasmodiophorid clade and five within the phagomyxids (Neuhauser et al. 2014). Phytomyxid biodiversity is still understudied (Bass et al. 2018; Neuhauser et al. 2014), and current data indicate that the diversity of this group of parasites is relatively low compared to their free‐living (Berney et al. 2013) and parasitic relatives (Hartikainen et al. 2014a; Hartikainen et al. 2014b; Ward et al. 2016; Ward et al. 2018). Phytomyxea are regularly detected in environmental sequencing studies, but do not get systematic attention in those studies because they are rare in terms of reads and there are usually few operational taxonomic units recovered (Ciaghi, S., Berney, C., Romac, S., Mahé, F., de Vargas, C., Jaillon, O., Kirchmair, M., Bass, D. & Neuhauser, S., unpubl. observ.). However, phytomyxids have been found in all habitats where their hosts are abundant, and DNA‐based studies have identified phytomyxid sequences globally in soils and rhizosphere (Legrève et al. 2002; Neuhauser et al. 2014; Sapp et al. 2018; Urich et al. 2008), but also in more extreme habitats such as the sea ice and open water in the Baltic Sea (Majaneva et al. 2012), moss pillars from a lake in Antarctica (Nakai et al. 2012), mangrove ecosystems (Zhu et al. 2018) or anoxic marine sediments (Takishita et al. 2007). More recent analyses of functional traits of cercozan and endomyxan parasites in the soil showed distinct impacts of phytomyxids in the rhizosphere (Fiore‐Donno et al., 2019). Although the ecological roles of phytomyxids in the soil and for shaping community structures start to receive attention, we are still lacking voucher sequences of many described and well‐characterised species, especially those infecting hosts without economic importance to add ecological context to eDNA sequences.

Taxonomy of phytomyxids is still rooted in the traditional morphotaxonomic concepts. Those are mainly based on symptoms of the host (hypotrophies or no hypertrophies) and the arrangement of the resting spores. Especially species of plasmodiophorids that do not form hypertrophies are discussed to play major roles in the rhizosphere (Bass et al. 2018; Fiore‐Donno et al., 2019), but data on those are still sparse in terms of available voucher sequences. Nongalling plasmodiophorids are (mainly) placed within the genera *Polymyxa* and *Ligniera*. Ledingham (1939) described Polymyxa graminis as different from Ligniera (and other plasmodiophorids) by the “formation of septate zoosporangia with conspicuous tubes for zoospore discharge.” Karling (1968) called the genus Ligniera a “convenient dumping ground for species which cause little or no hypotrophy and develop cystosori of indefinite shape, size and structure.” Whether *Ligniera* and *Polymyxa* are independent genera or not has been discussed repeatedly (Braselton 1989; Braselton 1995). Karyotypic analysis of *L. verrucosa* supports *Ligniera* as an independent genus and distinct from *Polymyxa* (Braselton 1989). However, *Ligniera verrucosa* for which extensive morphotaxonomic studies are available (Barr 1979; Miller et al. 1985) is morphologically clearly distinct from other *Ligniera* species. The type species of *Ligniera* is *L. radicalis* Maire et Tison, which is synonymous to *L. junci* (Basionym*: Sorosphaera junci;* Cook (1926)). For *Ligniera junci*, no detailed morphological studies based on ultrastructure are available; however, *L. junci* is the only species of this genus for which 18S rRNA information is available. Based on these rRNA data, the genera *Polymyxa* and *Ligniera* are closely related (Neuhauser et al. 2014). With more and more sequences of phyotmyxids becoming available, the morphological concepts which were used to determine genera and species lead to poly‐ and paraphyletic clades in molecular phylogenies (Neuhauser et al. 2014): *S. nasturtii* is genetically distant from *S. subterranea* and *Plasmodiophora* in its current concept is polyphyletic as well.

Aim of this study was to expand our understanding of phytomyxid diversity by collecting and analysing targeted samples from a diverse set of habitats across the globe. At the same time, we were aiming to amend problematic issues of phytomyxid taxonomy with increased taxon sampling. We were able to identify 40 novel environmental rRNA sequences that grouped within known genera but also formed two novel, so far undescribed environmental clades. During the sampling campaign, we also could find *L. verrucosa* which allowed us to generate the first voucher sequence of this species which fell into one of the new clades identified by this study. The expanded dataset also allowed us to address some of the open and debated taxonomic issues, although some of the problems must remain unsolved till more data are available or a consent on nomenclatural problems can be found.

## Material and Methods

### eDNA (environmental DNA) screening

Samples were collected between 2012 and 2018 at different locations in Austria, the UK, Italy and South Africa (Table 1 and Table S1).

**Table 1:** Sampling sites of eDNA samples

Sample sites/location	GPS coordinates	Date	Type
Wildlife garden NHM, UK	51.496035, −0.178467	10.4.2012	Soil/pond
Loch Eck, Scotland	56.0810128, −5.0690055	16.9.2012	Substrate
Pooled samples from Dervent water Keswick, UK	54.5832386, −3.1653003	12.9.2012	Water
Lohbach, Tirol, Austria	47°16′4″°N 11°20′42″°E	09.04.2013, 28.05.2013	Water, water cress
Field, Lohbach, Tirol, Austria	47°26′75″°N 11°34′11″°E	23.04.2018	Festuca pratensis
Field, Lohbach, Tirol, Austria	47°26′75″°N 11°34′11″°E	23.04.2018	Urtica dioica
Rotmoos Valley, Tirol, Austria	46°49′21″°N 11°02′55″°E to 46°50′36″°N 11°01′33″°E	10.07.2013	Soil
Brandis Waal, Italy	46.5915485, 11.1566942	12.7.2013	Water
Oxford garden, England	51.7505718, −1.255853	10.4.2012	*Taraxacum* sp.
South Africa	Ref Neuhauser 2014	12/2011	Soil
Etsch, Italia	46.608967, 11.1802445	10.07.2013	Water
Juncus effuses, Lake District, England	54.5832386, −3.1653003	13.4.2012	*Juncus* sp.
Moss sample, Lake District, England	54.5832386, −3.1653003	13.4.2012	Moss
Moss sample, Lake District, England	54.5832386, −3.1653003	13.4.2012	Moss
Fort William, Scotland	56.786273, −5.155318	18.9.2012	Beach/moss
Whitstable, England	51°21′41.2″N 1°01′23.4″E	15.4.2012	Beach/marine
Berglsteiner See, Tirol, Austria	47°28′21″°N 11°54′46″°E	25.04.2013, 15.06.2013	Moss
Berglsteiner See, Tirol, Austria	47°28′21″°N 11°54′46″°E	25.04.2013, 15.06.2013	Sediment
Field, Lohbach, Tirol, Austria	47°26′75″°N 11°34′11″°E	23.04.2018	*Veronica persica*

#### DNA extraction and 18S rRNA sequencing

DNA was extracted from root samples. Rinsed root or moss samples were microscopically screened for phytomyxid infections. When typical plasmodiophorid structures were present, infected roots were collected in 1.5 ml Eppendorf tubes (“phytomyxid isolates”). When no plasmodiophorid structures were found, the root system was randomly subsampled for DNA extraction. Phytomyxid isolates and root samples were homogenised using a FastPrep‐24™ 5G (MP Biomedicals, Heidelberg, Germany) and extracted with the DNeasy Plant Mini Kit (Quiagen, Hilden, Germany) following the manufacturer instructions with the modification that 60 µl instead of 100 µl Buffer AE was used to elute the DNA.

Soil samples were extracted using the PowerSoil Kit (Qiagen) according to the manufacturer’s instructions.

Water samples were filtered onto polycarbonate filters (0.4 µm, 47 mm, Millipore Isopore) in triplicates with a volume of 350 ml each. The DNA extraction was based on a 50‐50‐50 buffer‐chloroform/phenol method from the Laboratory for Environmental Pathogens Research Department of Environmental Sciences, University of Toledo (Sigler 2004).

All DNA samples were stored at −20 °C until they were further processed. All DNA samples were tested with either fungal‐ or cercozoan‐specific PCR primers to test whether the DNA extracts were okay. Different phytomyxid‐specific PCR primer combinations were used on all samples (Table 2), and positive (*P. graminis*) and negative controls (a PCR without adding DNA) were included in each set of reactions.

**Table 2:** PCR Primers: Primer combinations, annealing temperature [°C], elongation time [min], the expected size of the PCR product [bp] and the number of cycles are given

Forward primer	Primer sequence [5′–3′]	rDNA region	Reverse primer(s)	Primer sequence [5′–3′]	Annealing [°C]	Elongation [min]	Cycles	Size [bp]
s4f	GGCAGCAGGYGYGHAAATIRYCCA	SSU	C9rPhyt	GGAATTCCTCGTTGGTGCG	65	01:30	33	1,500
s6f	GAGGRNAAGYCTGGTGCCAGCASC	SSU	V7rpT5	CYGWCAGTCCCTCTAAGAAGTCGA	60	01:30	39	800
			V7rPhag	ACACCGAYMGTCCYTCTCAATCCT				
			V7rNC9	CTAACACGCKGAGGTCTCGTG				
PlasSSUF1	TCAGTGAATCTGCGGATGGC	SSU	PlasSSUR4	GGTGCSKCKAGRTVCAAGAGGC	60	01:45	35	1,500
PlasSSUF2	TGGATGTACGAGAGTACTACATGG	SSU	PlasSSUR3	CGTTGAACCTAGCATTGTAGCG	60	01:45	35	1,500
Pre3NDf	CAGCAGGCGCGCAAATTACC	SSU	1256r‐CO	GCACCACCACCCAYAGAATCAAGAAAGAWC	52	00:40	35	850
ITS 1	CAYAGAATCAAGAAAGAWCTTC‐	ITS1‐5.8S‐ITS2	ITS 4	TCCTCCGCTTATTGATATGC	52	00:40	35	450
s4f	GGCAGCAGGYGYGHAAATIRYCCA	SSU	sB2phy	CCTTGTTACGACTTCTYCTTCYTC	65	01:30	33	1,500
			sB2end	CCTTGTTACGACTTCTCCTTCCTC				
*V4fmix: (V4fEnd +	GTGCCAGCAGCCGCGGTAAYA	SSU	*s1256r mix: (1256R‐Ph +	CACYACCCATAGAATCAAGAAAGAGCTKCA	67.5	01:00	39	800
V4fEuk)	CCAGCASCCGCGGTAAYWCC		1256R‐PI)	CACCACCGAAGTGATCAAGAAAGAKCTKCA				

Primers with * were used for nested PCR.

Each PCR mix (30 µl) included final concentrations of 0.2 mM dNTP mix (Fermentas, Waltham, MA), 1 µM of each primer, 3 mM MgCl_2_ (Promega, Madison, WI), 1× GoTaq flexi buffer (Promega), 2 mg/ml BSA (Sigma‐Aldrich, Gillingham, UK) and 0.25 U GoTaq DNA polymerase (Promega). 2 µl DNA extract was used for each reaction.

PCR conditions were 95 °C for 3 min, followed by 33 cycles of 95 °C for 30 s, 52–65 °C (depending on the primers, Table 2) for 30 s and 72 °C for 90 s. This was followed by a 10‐min final elongation step at 72 °C. PCR products were visualised on a 1% agarose gel (Biozym Scientific GmbH, Hessisch Oldendorf, Germany) stained with 0.1 µl of SYBR Safe (Thermo Fisher Scientific, Waltham, MA) per 30 ml.

PCR products from material with microscopically confirmed infections were purified using the GFX PCR DNA and Gel Band Purification Kit (Sigma‐Aldrich) and sequenced at Microsynth Austria. All other PCR products that showed the expected length were purified using a PEG precipitation protocol (Neuhauser et al. 2014) (www.mcdb.lsa.umich.edu/labs/olsen/files/PCR.pdf) and were sequenced at Macrogen Europe after cloning.

#### Cloning of PCR products

To improve the efficiency of the TA‐cloning, PCR products were A‐tailed: 10 µl of purified PCR product was incubated at 72°C for 15 min with 1× PCR buffer, 0.05 mM dATP and 0.25 U Taq Polymerase. These products cleaned via Sephadex columns (2% Sephadex) and cloned immediately using the “Thermo Scientific™ InsTAclone™ PCR Cloning Kit” (Thermo Fisher Scientific) according to the manufacturer’s instructions into chemically competent *Escherichia coli* JM 107. From each sample, 6–18 colonies were picked and added to the PCR mix (colony PCR). The PCR mix (20 µl) contained final concentrations of 1 × Taq buffer (Fermentas), 0.2 mM dNTP (Fermentas), 1.5 mM MgCl_2_ (Promega), 0.3 µM of each primer M13r/M13f and 0.25 U Taq Polymerase (Fermentas) or 0.25 U GoTaq Polymerase (Promega). The DNA amplification was performed by 94 °C for 2 min initial denaturation, followed by 30 cycles with 30 s at 94 °C, 30 s at 45 °C and 72 °C for 1 min.

### Phylogenetic analyses

Sequences were blasted (blastn) against GenBank (http://www.ncbi.nlm.nih.gov/genbank) and the pr2 database (http://ssu‐rrna.org) to identify phytomyxea. Sequences were curated manually in BioEdit version 7.2.5 (Hall 1999). The 18S rRNA sequences generated in this study (Table S1) were deposited in NCBI GenBank under the accession numbers MN170945–MN179084. The sequence of *L. verrucosa* and the newly generated eDNA sequences were aligned to an existing dataset (Neuhauser et al. 2014). The datasets were analysed using the Geneious R9.1.5 (http://www.geneious.com; (Kearse et al. 2012)) plugins for RAxML (7.2.8, Stamatakis 2006), PHYML (Guindon et al. 2010) and MrBayes (3.2.6, Huelsenbeck and Ronquist 2001). RAxML settings were as follows: GTR + CAT model with rapid bootstrapping (100 replicates) and search for the best scoring ML tree, start from complete random tree. MrBayes analyses were run with two sets of four chains for 1 million generations, a subsampling frequency of 1,000 and a burnin of 10% using the GTR + G + I model. PHYML settings were as follows: GTR with chi‐square statistics, four substitution rate categories and the settings to optimise topology/length/rate and the BEST topology search option selecting the best topology of NNI and SPR search. Trees were annotated using TreeGraph2.14.0‐771 beta (Stöver and Müller 2010) and Adobe Photoshop CS5.

#### Sampling

Material examined: *Ligniera verrucosa* was sampled at a park in Innsbruck Austria, Tyrol, 47.1545, 11.2340), in roots of *Veronica persica* collected on the 4.4.2018, 7.5.2018 & 25.5.2018. The infected roots were rinsed with water and examined under a light microscope. Roots of *Veronica persica*, as well as roots from possible alternative host (*Glechoma hederacea*, *Festuca pratensis*, *Alliaria petiolata*, *Taraxacum officinale*, *Urtica dioica* and *Ranunculus* sp.), were microscopically examined for the presence of *Ligniera verrucosa* and other plasmodiophorids. The plasmodiophorids were identified morphologically by their resting spores. For microscopic identification, the plasmodiophorid monography by Karling (1968) was used. Reference microscope slides of *L. verrucosa* (IB20200003), *P. graminis* (IB20200004) and *L. junci* (IB20200002) have been deposited at the herbarium of the University of Innsbruck.

#### Light microscopic investigations

For light microscopy, a Nikon Optiphot 2 light microscope was used. Pictures were made with the Digital Sight DS 5M (Nikon, Kawasaki, Japan) and the NIS Elements D 3.0 software (Nikon). Linear adaptions to brightness and contrast, resizing, cropping and assembly of the figures were done with Adobe Photoshop CS5. Photographs were cleaned up for aesthetic reasons, without altering areas of scientific significance. Unmodified images are deposited at figshare: https://doi.org/10.6084/m9.figshare.12587624.v1.

## Results

### Biodiversity

**Figure 1:**
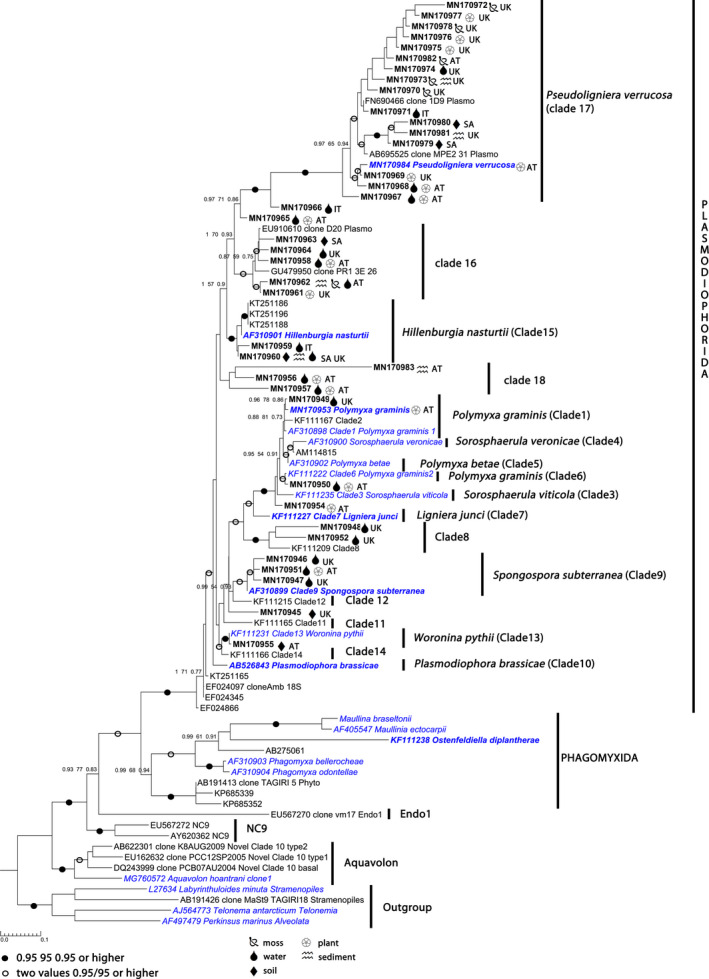
Phylogenetic tree (PHYML) of plasmodiophorid 18SrRNA sequences. Isolates are shown in blue, polyphyletic genera are highlighted using bold letters. The clades are numbered according to (Neuhauser et al. 2014) newly identified clades are numbered subsequently. 87 sequences 796 positions. Chi^2^ statistics, RAxML bootstraps and posterior probability values are shown if 0.70 or higher.

Overall, 40 new plasmodiophorid 18S rRNA sequences from 20 environments and from two isolates (*L. verrucosa* and *P. graminis*) were generated in this study (Table S2). Fifteen of those sequences belonged to characterised species or previously described environmental clades (Neuhauser et al. 2014). Another 20 of these sequences formed two novel, not yet characterised clades belonging to the Plasmodiophorida (Fig. 1). Five of our new sequences did not branch with any defined clade.

Both phytomyxean orders Plasmodiophorida and Phagomyxida were strongly supported in the phylogenetic analysis (Fig. 3, chi‐square statistics/RAxML bootstraps/posterior probability values 1/100/1 for Plasmodioporida and 0.99/68/0.94 for the Phagomyxida). Seventeen well‐supported clades could be resolved in Plasmodiophorida, two of them new, which were named clade 17 and 18. Clade 16, which was previously defined as an environmental clade comprising three sequences (Neuhauser et al. 2014), was the second clade with clearly increased biodiversity in this study. Five novel lineages from five habitats were added to this clade, which now includes sequences from moss, plant, soil and water samples. Clade 16 still comprises only environmental sequences and is well supported (1/85/1).

Clade 17 consisted of 19 18S rRNA lineages from 13 habitats—most of the habitats were moss‐dominated. The sequence of *Ligniera verrucosa* clustered with these sequences. Due to the new nomenclatural combination, later in this manuscript we refer to this clade as the *“Pseudoligniera verrucosa* clade.” The *P. verrucosa* clade was strongly supported by chi‐square statistics, RAxML bootstraps and posterior probability values (1/100/1) (Fig. 1). Within this clade, there was one isolate, the rest were environmental sequences originating from plant, moss and water samples from Austria, Italy, South Africa and the UK (Table S1/Fig. 3) as well as two sequences from GenBank (FN690466.1 and AB695525.1) which originated from sea ice and open water in the Baltic Sea (Majaneva et al. 2012) and aquatic moss pillars from a freshwater lake in East Antarctica (Nakai et al. 2012).

Clade 18 consisted of three sequences originating from the Lohbach and Berglsteinsee in Tirol, Austria. This clade had only weak support (0.98/27/0.84) but was distant from all other clades why we decided to refer to it by the informal name Clade 18.

The addition of sequences from these two species made the genus *Ligniera* polyphyletic, necessitating the removal of *L. verrucosa* from the genus *Ligniera* and rename it as *Pseudoligniera verrucosa* as discussed in the taxonomic summary. *L. verrucosa* branched within the newly described Clade 17 while *L. junci* grouped with Clade 7 (Figure S1 highlighted in red; Fig. 3). *L. junci* formed a well‐supported subclade (Clade 7) on the basis of the clade containing with *P. graminis* (Clade 1, including the 18S sequence of the novel isolate), *Polymyxa betae* (Clade 5), *Sorosphaerula viticola* (Clade 3) and *Sorosphaerula veronicae* (Clade 4). Clade 4 also contained MN170954, an 18S rRNA sequence associated with the roots of *Urtica dioica* at the site where *L. verrucosa* was found. Although the genera *Polymyxa* and *Sorosphaerula* are polyphyletic in the tree presented here, the support and branching order are not very strong. Therefore, we decided that in the sense of taxonomic stability taxonomic amendments in these genera are not justified based on the presented dataset.

The genera *Spongospora* and *Plasmodiophora* were also rendered polyphyletic by the analyses including our new data. Within the genus *Spongospora* (*S. subterranea* Clade 9, *S. nasturtii* Clade15), the species *Spongospora nasturtii* is excluded from this genus and renamed *Hillenburgia nasturtii* as described in the taxonomic summary (Fig. 1 and Fig. S1). The two species of the genus *Plasmodiophora* belong to different orders. The soil‐borne brassica infecting *Plasmodiophora brassicae* belongs to the Plasmodiophorida while the marine seagrass parasite *Plasmodiophora diplantherae* belongs to the Phagomyxida (Fig. 1 and Fig. S1) requiring the latter to be reverted to its original name *Ostenfeldiella diplanterae* Ferd. & Winge (Ferdinandsen and Winge 1914).

### Morphological and molecular characterisation

#### *Ligniera verrucosa* Maire and Tison, 1911

**Figure 2:**
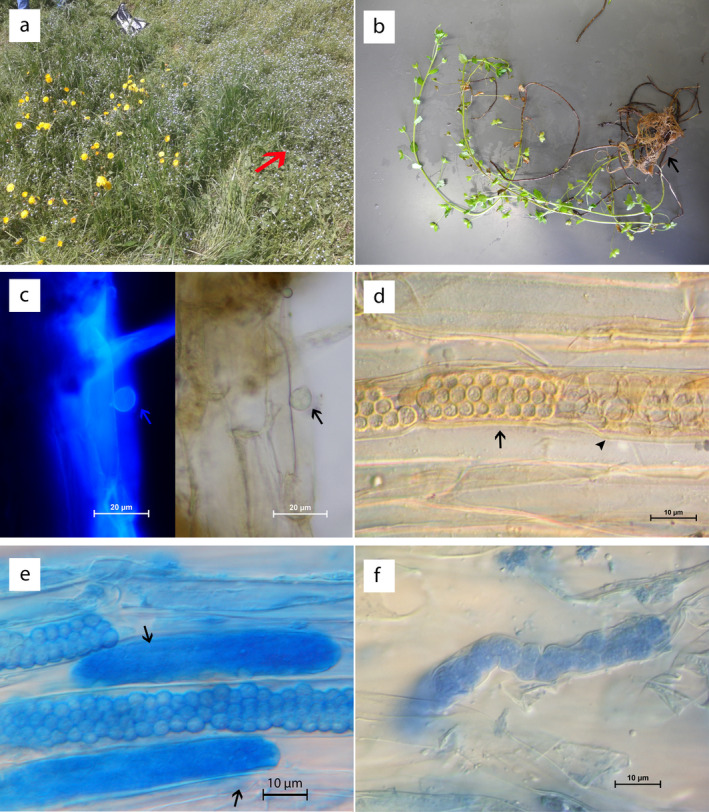
*Pseudoligniera verrucosa* comb. nov. (a) Sampling site of *P. verrucosa* with *Veronica persica* (arrow) in Innsbruck, Austria. (b) *V. persica*, infected with *P. verrucosa* with infected roots (arrow). (c) *P. verrucosa* zoospore (arrow) infecting the roots of *V. persica*. Calcofluor White staining, left panel and brightfield image of the right panel. (d) Resting spores aggregated in a characteristic sporosori (arrow) next to empty zoosporangia (arrowhead). (e) Plasmodia (arrows) and sporosori of *P. verrucosa*. (f) Plasmodia of *P. verrucosa*. Scale bars: 20 µm (c), 10 µm (d–f).

*Ligniera verrucosa* was found in the roots of *Veronica persica* (Fig. 2), but not in the roots of other plants collected nearby, neither microscopically nor by using specific primers. The resting spores were ovoid to ellipsoidal, (2.1) 3.1 ± 0.5 (4.1) µm (*n* = 61) diam. with hyaline, verrucose walls. The resting spores aggregated into sporosori differing in size and shape (Fig. 2d, e). Plasmodia were observed in roots (Fig. 2e, f), and zoosporangia were present in roots as well as in root hairs (Fig. 2d). In one instance, a putative zoospore attached to the root cortex was observed (Fig. 2c).

**Figure 3:**
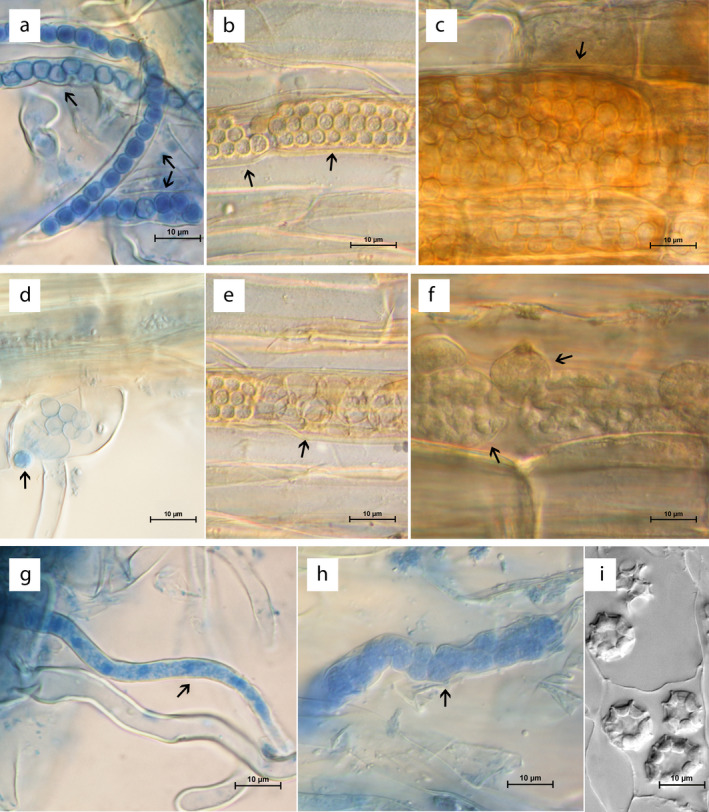
Comparison of Ligniera junci (a, d, g), *Pseudoligniera verrucosa* (b, e, h), and *Polymyxa graminis* (c, f). (a): Resting spores of *L. junci* arranged linearly in root hair (arrows), (b) resting spores of *P. verrucosa* forming characteristic sporosori. (c) *P. graminis* resting spores. (d): Discharged zoosporangia of *L. junci* with one zoospore (arrow), (e) Empty zoosporangia of *P.verrucosa* (arrow). (f) Zoosporangia of *P. graminis*. (g) Plasmodia of *L. junci* in root hair of *Juncus* sp. (h) *P. verrucosa* plasmodium undergoing differentiation. (j) *S. viticola* resting spores. Scale bars: 10µm (a – h). For the comparison of *Pseudoligniera verrucosa* with other plasmodiophorid species we reused the most characteristic images of *P. verrucosa* from Figure 2 to ensure high quality images with all the important features of the species.

#### *Ligniera junci* (Schwartz) Maire and Tison, 1911

Resting spores of *Ligniera junci* were found in root hairs of *Juncus* sp. Resting spores were subspherical to ovoid, (2.9) 3.8 ± 0.5 (4.8) × (2.7) 3.8 ± 0.5 (4.7) µm (*n* = 37) with hyaline, smooth walls. The resting spores were aggregated into sporosori of linear shape, which was likely influenced by the shape of the root hairs (Fig. 3a). Zoosporangia and a zoospore were observed in a root hair (Fig. 3d). Plasmodia were found in root hairs of *Juncus* sp. (Fig. 3g).

#### *Polymyxa graminis* Ledingham, 1939

Resting spores and zoosporangia of the plasmodiophorid *Polymyxa graminis* were identified in the roots of *Festuca pratensis* from the same plot where *L. verrucosa* was found (Fig. 3c–f).

Resting spores were subspherical to ovoid, (3.6) 4.8 ± 0.6 (6.1) µm (*n* = 30) diam. (Fig. 3c) and formed loose sporosori. Neuhauser and Kirchmair (2009) measured resting spores with the size of (3.8–)5.3 ± 0.6(−7.4) × (3.2‐)4.6 ± 0.6(−6.3) μm in diam.; Q = (1)1.2 ± 0.1(−1.6); *n* = 59, Karling (1968) mentions a resting spore size of 4–7 μm, while Ledingham (1939) reports a resting spore size of 5–7 μm in diameter. Enveloped zoosporangia of irregular shape filled with zoospores were found in the roots (Fig. 3f).

## Discussion

### Molecular data

Traditionally, discrimination of phytomyxid species and genera was based on morphological criteria like resting spore and sporosori size and shape, together with host specificity (Bulman and Braselton 2014; Karling 1968). However, these morphological species and genus concepts are only partly reflected in earlier phylogenetic analyses (Neuhauser et al. 2014) and those presented in this study. Three genera (*Ligniera*, *Plasmodiophora* and *Spongospora*) were polyphyletic in our new analyses. The genera *Polymyxa* and *Sorosphaerula* are morphologically well separated: *Polymyxa* spp. form sporosori of indefinite size and shape (spherical, globose to polyhedral) without a membrane (Karling 1968; Ledingham 1939), while the genus *Sorosphaerula* is defined by their spherical to ellipsoidal hollow sporosori (Cook 1933). In our phylogenetic tree, *P. gramins*, *P. betae*, *S. veronicae* and *S. viticola* formed a well‐supported clade, similar to previous studies (Bulman et al. 2001; Neuhauser et al. 2014). However, the four species within this cluster are not well resolved as phylogenetically distinct entities. A more complete dataset with more sequences spanning the full 18S and 28S rRNA array will be required to resolve relationship and phylogeny within the *Polymyxa/Sorosphaerula* clade.

In the Clades 18, 16 and particularly clade 17 (*Pseudoligniera verrucosa* clade), novel environmental sequences were added in this study, expanding the known diversity. Clade 16 was already established (Neuhauser et al. 2014) based on environmental sequence originating from a submerged sinkhole (EU910610, (Nold et al. 2010)) and a peat bog (GU479950). New sequences from different habitats (water, plants, moss and soil) in South Africa, Scotland, England and Austria added new diversity to clade 16. Although clade 16 does not include any characterised taxa, the presence of the same sequence type in independent studies and from geographically well‐separated areas suggests that clade 16 very likely is a parasite with a wide range of hosts.

The *Pseudoligniera verrucosa* clade (clade 17) was well supported and clearly distinct from other clades (Fig. 1). This clade appears to be diverse in sequences based on environmental sequences and contains one isolate, *Ligniera verrucosa*. The sequences of clade 17 were found mainly associated with plants (including mosses) but also with soil, and fresh or brackish water collected in Austria, Scotland, England, South Africa and Antarctica. Plasmodiophorids are currently thought to be exclusively nonmarine (Neuhauser et al. 2014), and sequences of clade 17 were mainly found in freshwater. However, one of the GenBank sequences in clade 17 has marine water and ice samples stated as origin (FN690466). This sequence was deposited in GenBank as part of the study by (Majaneva et al. 2012) where different forms of sea ice, including sea ice attached to land were sequenced. It was not possible to determine from which of the samples this clone derived, so a marine origin of the sequence cannot be verified. Therefore, it remains to be proven whether or not there are plasmodiophorids which can infect marine hosts, and whether this sequence represents a parasite infecting an organism in arctic moss pillars or its spores arrived in this environment randomly (Caliz et al. 2018; Eisenhofer et al. 2019; Rennie et al. 2015). Noticeably, many of the *Pseudoligniera*‐clade sequences originated from moss‐dominated environments in the UK and Austria, so it is likely that the organisms belonging to the sequences are parasites of a host that is present in moss‐related environments. Studies specifically targeting the potential hosts within these environments will be needed to identify this parasite and its hosts.

By sampling water bodies, eDNA lineages belonging to various previously characterised clades were found (Fig. 1). It is known that the clubroot pathogen *P. brassicae* can be transmitted via water bodies like other plant pathogens (Hong and Moorman 2005; Rennie et al. 2015; Zappia et al. 2014). Here, we were able to detect sequences from environmental samples belonging to the *P. verrucosa* clade, Clade 16, *H. nasturtii*, Clade 18, *P. graminis* and *S. subterranea* in water bodies in Austria, Italy and the UK. These data are the first to provide evidence that transmission via water bodies might be a relatively common means of dispersal for many plasmodiophorid species. We analysed only relatively small amounts of water in this study (350 ml per sample). Therefore, screening of water bodies might provide a relatively easy method to analyse the overall biodiversity in larger areas (Hong and Moorman 2005); however, this needs to be tested in future studies.

Finding easy, fast and convenient methods to screen many habitats over short periods of time are of interest as such methods will allow to use tight time courses and to include spatial pattern into biodiversity studies. This is important as some phytomyxids have seasonal pattern. During this study, *L. verrucosa* was only detected for a short time‐window in spring in the roots of the host, when its host, *Veronica persica*, had its first and most prominent bloom. Although *V. persica* was present and also flowering at the site later in the year, we were not been able to find any resting spores in the roots of this host plant other than in April/May. Based on observations of other plasmodiophorids (Karling 1968; Neuhauser et al. 2011a; Neuhauser et al. 2011b), such seasonal patterns could hamper the identification of novel biodiversity. Seasonal patterns are common for obligate parasites, especially those infecting short‐lived hosts (Garvetto et al. 2019; Scholz et al. 2016; Torres‐Beltran et al. 2018), so approaches and survey designs permitting fast and standardised seasonal screening of selected habitats could increase our knowledge of phytomyxid diversity significantly in the future.

### Morphological data

#### Comparison of nongalling Plasmodiophorids: Ligniera verrucosa, Ligniera junci Polymyxa graminis and Sorosphaerula viticola

The resting spores of *L. verrucosa* differ in their shape from the resting spores of *L. junci*. While the walls of the resting spores of *L. verrucosa* are verrucose (Fig. 2d), resting spores of *L. junci* have hyaline and smooth walls (Fig. 3a, b). Resting spores of *L. verrucosa* are usually found in roots while the majority of resting spores of *L. junci* are found in root hairs. The resting spores of *L. verrucosa* form a tightly packed sporosori while the resting spores of *L. junci* are smaller and looser associated with a sporosori (Fig. 3a–c). *P. graminis* differ by their enveloped zoosporangia with an exit tube and their bigger resting spores from *L. junci* and *L. verrucosa*. The resting spores of *Sorosphaerula viticola* are arranged in hollow spheres (Fig. 3j; Kirchmair et al. 2005). The species are discriminated by their host ranges: *L. verrucosa* is found in *Veronica spp*., *L. junci* in *Juncus spp*., *P. graminis* in different *Poaceae* and *Sorosphaerula viticola* in *Vitis* spp.

*Ligniera junci* and *L. verrucosa* can be discriminated through their different resting spore morphology and their host range. The warty structure of the resting spores of *L. verrucosa* differs from the smooth resting spores of all *Ligniera* species (Fig. 3a–c, (Karling 1968), which together with phylogenetic analyses supports *L. verrucosa* belonging to an yet undescribed genus separate from the *Ligniera*. Karyotypic analysis, as well as ultrastructure analysis (especially of *Ligniera junci*) may help to get a more complete picture of species defining features of phytomyxids.

## Taxonomic Summary

In the phylogenetic tree (Fig. 1), eight of twelve currently recognised genera within the Phytomyxea are included. Three genera (*Ligniera*, *Spongospora* and *Plasmodiophora*) are polyphyletic requiring the nomenclatural changes below and shown in Fig. 1.

### ***Ligniera*** Maire and Tison

The genus *Ligniera* was first described by Maire and Tison (1911a) for plasmodiophorids which do not cause hypertrophies in their hosts. The genus *Ligniera* originally included three species, *L. verrucosa*, *L. junci* and the type species *L. radicalis*. Maire and Tison (1911b) mentioned the similarity between *L. junci*, originally described as *Sorosphaera junci* (Schwartz 1910) and *L. radicalis*. This similarity was also noted by Cook (1926) who concluded that the only difference between the two species is the size of the resting spores. Guyot (1927) suggested that those two species are conspecific. Cook (1933) followed the arguments of Guyot (1927) and synonymised *L. radicalis* with *L. junci*. As the older name, the epithet “junci” has priority over “radicalis.” This synonymy was accepted and adopted by Karling (1968). *L. junci* was further analysed and re‐described by Neuhauser and Kirchmair (2009). In the phylogeny presented here, the genus *Ligniera* is polyphyletic as *L. verrucosa* clustered in a different clade from *L. junci*. Consequently, *L. verrucosa* must be excluded from *Ligniera*.

### *Ligniera junci* (Schwartz) Cook

**Basionym:** *Sorosphaera junci* Schwartz, 1910.

Karling (1968) mentioned a resting spore size of 4–7 µm, Neuhauser and Kirchmair (2009) a resting spore size of (4.5‐)5.4 ± 0.6(−6.8) × (3.3‐)4.6 ± 0.5(−5.6) μm; *Q* = (1.0‐)1.2 ± 0.1(−1.6); *n* = 49.

**Diagnosis (Ivimey Cook** 1926): “Sporis levibus 5–7 µ diam., in acervulos saepe cavos conjunctis. Habit in radicibus plantarum aquatilium et palustrium.”

### ***Pseudoligniera* gen. nov.** Neuhauser, Hittorf, Kirchmair

**Type species:** *Pseudoligniera verrucosa* (Basionym: *Ligniera verrucosa* Maire and Tison, 1911b)

### ***Pseudoligniera verrucosa* (Maire and Tison)** Neuhauser, Hittorf, Kirchmair

Resting spores ovoid to ellipsoidal, with thick, verrucose cell walls. Forming cytosori of indefinite size and shape. Can be found in roots of *Veronica spp*., *Beta vulgaris*,*Bromus* sp., *Chenopodium album* and *Festuca* sp. ((Karling 1968) Guyot 1927).

**Basionym:** *Ligniera verrucosa* Maire and Tison, 1911b

(Original diagnosis (Maire and Tison, **1911b):** “Sporis crasse verrucoses, 4–5 µ diam., in acervulos plenos conjunctis.”

### *Plasmodiophora* Woronin

The genus *Plasmodiophora* was described by Woronin (1878) with *P. brassicae* as type species. Members of the genus *Plasmodiophora* cause galls or hypertrophies in the host tissue. These plasmodiophorids do not form sporosori, and the resting spores lie individually in the host cell (Karling 1968). Karling (1968) lists six species in the genus *Plasmodiophora*, including *P. brassicae* and *P. diplantherae*. Based on molecular data (Fig. 1 and Fig. S1), *P. diplantherae* has to be excluded from *Plasmodiophora*.

### ***Ostenfeldiella*** Ferdinandsen and Winge

*Plasmodiophora diplantherae* was first described by Ferdinandsen and Winge (1914) as *Ostenfeldiella diplantherae* and formed with this species the genus *Ostenfeldiella*. Cook (1933) suggested placing *O. diplantherae* into the genus *Plasmodiophora* because of the shape and the position of the resting spores. Braselton and Short (1985) however found significant differences in the number of synaptonemal complexes between *P. diplantherae* and *P. brassicae*. Neuhauser et al. (2014) showed that *P. diplantherae* branches in Phagomyxida and is phylogenetically very distant from *P. brassicae*, which branches in Plasmodiophorida. Because *P. brassicae* is the type species of the whole group, *P. brassicae* should remain in the genus *Plasmodiophora* and the original name *Ostenfeldiella diplantherae* should be used for *P. diplantherae*.

**Original diagnosis (Ferdinandsen and Winge** 1914 **):** “Genus caulicolum, submarinum Plasmodiophoracearum, Plasmodio‐phorae propius accedens, colore autem saturato sporarum nee non modocrescendi proprio satis distinctum. Etymologia a cl. doctore C. H. Ostenfeld,fungi inventore, de studio plantarum maris vascularium optime merito.”

### *Ostenfeldiella diplantherae* Ferdinandsen and Winge

**Original diagnosis (Ferdinandsen and Winge** 1914 **):** “Myxamoebae uninucleatae in partibus merismaticis caulium hospitis nunc singulatim, nunc plures in cellula singula inventae, corticem interiorem solum infestantes. Ad basim internodii secundi myxamoebae plurinucleatae sese formare incipiunt, et in internodiis sequentibus sporosori, cellulas pluries auctas, 125–200 µ. diametro, totum implentes, iacent. Sporae globosae, 4–4½µ diametro, siccitate collabescentes, singulatim brunneolae, gregatim saturate brunneae, membrano satis crasso indutae, plasmate oleoso, flavescenti, refringenti farctae. In internodiis brevibus caulium ascendentium limo sepultorum *Diplantherae Wrightii* Aschers., qui aggressu fungi ad modum siliquae *Ornithopodis* sativi usque ad 3 mm. diametro intumescunt, ad litus insulae St. Crucis Indiae occidentalis (Leg. C. H. Ostenfeld).”

### *Spongospora* Brunchorst

The genus *Spongospora* was established by Brunchorst (1887) with spores arranged in hollow or irregularly channelled sporosori (Cook 1933). The genus *Spongospora* currently contains four species, with *S. subterranea* as type species (Karling 1968). *S. subterranea* was described by Wallroth (1842) as *Erysibe subterranea*. Lagerheim (1892) suggested renaming it as *S. subterranea*. Tomlinson (1958) differentiated between *S. subterranea f*. sp.*subterranea* and *S. subterranea f*. sp.*nasturtii*. The only difference noted between those two species was the host; microscopically they are indistinguishable (Karling 1968). Dick (2001) however raised the two special forms into the rank of separate species on the bases of host specificity, habitat differences and differences in sporangial stages. Early 18S phylogenies by Bulman et al. (2001), Grenville and Clarkson (2002), and Qu and Christ (2004) support *S. subterranea* and *S. nasturtii* as separate species. The polyphyly of the genus *Spongospora* (Fig. 1 and Fig. S1) was already mentioned by Neuhauser et al. (2014) but without any taxonomic adaptions. *Spongospora subterranea* is the type species of the genus *Spongospora*; therefore, we rename *S. nasturtii* to *Hillenburgia nasturtii*.

### ***Hillenburgia* gen nov.** Neuhauser, Hittorf, Kirchmair

**Type species**: *Hillenburgia nasturtii (Wallr.) Neuhauser*,*Hittorf*,*Kirchmair* (Basionym *Spongospora subterranea* (Wallr.) Lagerh. f.sp. *nasturtii Tomlinson*)

**Etymology**: This genus is dedicated to Stephen Hillenburg, the creator of SpongeBob SquarePants, because of the spongy nature of the sporosori.

***Spongospora subterranea f. sp. nasturtii* Tomlinson Original diagnosis (Tomlinson** 1958 **)**: “Varians e forma parasitica *Nasturtium officinale* R.Br. et *N. officinale* R.Br. × *N*. *microphyllum* Boenn. ex Rehb. radices adunca et magna productus est. Typus: I.MI. 74293.

Differs from the type in parasitising *Nasturtium officinale* R.Br. and *N. officinale* R.Br. × *N. microphyllum* Boenn. ex. Rchb. causing root distortion and swelling. Type collection on *N. officinale* × *N. microphyllum*, Bere Regis, Dorset, May 1954, coll. J.A. Tomlinson.”

## Supporting information


**Figure S1.** Tree supporting the taxonomy presented in Fig. 1, only including sequences from isolates.

**Table S1.** New Sequences generated in this study.

**Table S2.** All Sequences used for phylogenetic trees in this study.
