# Supplementary material for: Revised Taxonomy and Expanded Biodiversity of the Phytomyxea (Rhizaria, Endomyxa)
Source: J Eukaryot Microbiol. 2020 Nov 8;67(6):648–59. doi: 10.1111/jeu.12817 (PMC7756720; doi:10.1111/jeu.12817)
Supplement: Supplementary file 1 — Figure S1. Tree supporting the taxonomy presented in Fig. 1, only including sequences from isolates. [file JEU-67-648-s001.pdf]

SUPPORTING INFORMATION

Revised Taxonomy and Expanded Biodiversity of the Phytomyxea (Rhizaria, Endomyxa) by Michaela Hittorf, Susanne Letsch-Praxmarer, Alexandra Windegger, David Bass, Martin Kirchmair, Sigrid Neuhauser

Figure S1. Tree supporting the taxonomy presented in Fig. 1, only including sequences from isolates.

Table S1: New Sequences generated in this study

Table S2: All Sequences used for phylogenetic trees in this study.

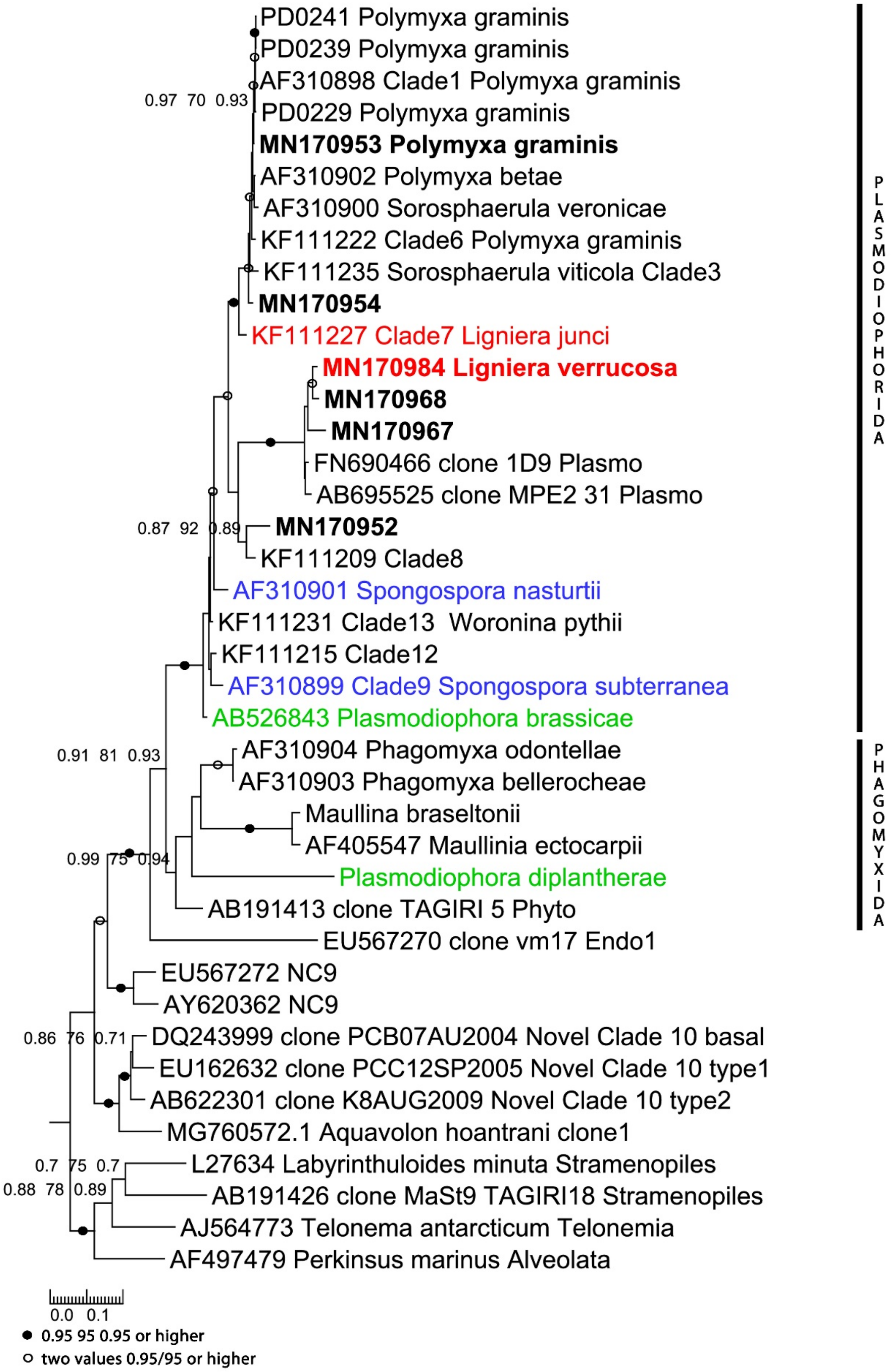

PD0241 Polymyxa graminis
PD0239 Polymyxa graminis
AF310898 Clade1 Polymyxa graminis
0.97 70 0.93
PD0229 Polymyxa graminis
MN170953 Polymyxa graminis
AF310902 Polymyxa betae
AF310900 Sorosphaerula veronicae
KF111222 Clade6 Polymyxa graminis
KF111235 Sorosphaerula viticola Clade3
MN170954
KF111227 Clade7 Ligniera junci
MN170984 Ligniera verrucosa
MN170968
MN170967
FN690466 clone 1D9 Plasmo
AB695525 clone MPE2 31 Plasmo
0.87 92 0.89
MN170952
KF111209 Clade8
AF310901 Spongospora nasturtii
KF111231 Clade13 Woronina pythii
KF111215 Clade12
AF310899 Clade9 Spongospora subterranea
AB526843 Plasmodiophora brassicae
0.91 81 0.93
AF310904 Phagomyxa odontellae
AF310903 Phagomyxa bellerocheae
Maullina braseltonii
AF405547 Maullinia ectocarpii
0.99 75 0.94
Plasmodiophora diplantherae
AB191413 clone TAGIRI 5 Phyto
EU567270 clone vm17 Endo1
EU567272 NC9
AY620362 NC9
0.86 76 0.71
DQ243999 clone PCB07AU2004 Novel Clade 10 basal
EU162632 clone PCC12SP2005 Novel Clade 10 type1
AB622301 clone K8AUG2009 Novel Clade 10 type2
MG760572.1 Aquavolon hoantrani clone1
0.7 75 0.7
L27634 Labyrinthuloides minuta Stramenopiles
0.88 78 0.89
AB191426 clone MaSt9 TAGIRI18 Stramenopiles
AJ564773 Telonema antarcticum Telonemia
AF497479 Perkinsus marinus Alveolata
PLASMODIOPHORIDA
PHAGOMYXIDA
0.0 0.1
● 0.95 95 0.95 or higher
○ two values 0.95/95 or higher
